# Multi-Scale Feature Fusion and Attention-Enhanced R2U-Net for Dynamic Weight Monitoring of Chicken Carcasses

**DOI:** 10.3390/ani16030410

**Published:** 2026-01-28

**Authors:** Tian Hua, Pengfei Zou, Ao Zhang, Runhao Chen, Hao Bai, Wenming Zhao, Qian Fan, Guobin Chang

**Affiliations:** 1 College of Animal Science and Technology, Yangzhou University, Yangzhou 225009, China; huatian0202@163.com (T.H.);; 2 College of Information Engineering, Yangzhou University, Yangzhou 225009, China; mz120240980@stu.yzu.edu.cn (P.Z.);; 3 Joint International Research Laboratory of Agriculture and Agri-Product Safety, The Ministry of Education of China, Yangzhou University, Yangzhou 225009, China

**Keywords:** deep learning, image segmentation, broiler carcass weights, weight prediction

## Abstract

In recent years, real-time monitoring of broiler chicken weight has become crucial for assessing growth and health status. Currently, obtaining weight data often relies on manual collection. However, this process is cumbersome, labor-intensive, and inefficient. This paper proposes a broiler carcass weight detection model based on deep learning image segmentation and regression to address these issues. The model first segments broiler carcasses and then uses the pixel area of the segmented region as a key feature for a regression model to predict weight. A custom dataset comprising 2709 images from 301 Taihu yellow chickens was established for this study. A novel segmentation network, AR2U-AtNet, derived from R2U-Net, is proposed. To mitigate the interference of background color and texture on target carcasses in slaughterhouse production lines, the Convolutional Block Attention Module (CBAM) is introduced to enable the network to focus on areas containing carcasses. Furthermore, broilers exhibit significant variations in size, morphology, and posture, which impose high demands on the model’s scale adaptability. Selective Kernel Attention (SKAttention) is therefore integrated to flexibly handle broiler images with diverse body conditions. The model achieved an average Intersection over Union (mIoU) score of 90.45%, and Dice and F1 scores of 95.18%. The regression-based weight prediction achieved an $R^{2}$ value of 0.9324. The results demonstrate that the proposed method can quickly and accurately determine individual broiler carcass weights, thereby alleviating the burden of traditional weighing methods and ultimately improving the production efficiency of yellow-feather broilers.

## Simple Summary

Utilizing computer vision technology to develop non-contact methods for broiler weight detection enables real-time monitoring of broiler weight changes. At present, there are very few reports, either internationally or domestically, on broiler carcass weight detection. In this study, a dynamic broiler weight detection model based on deep learning image segmentation is proposed. This model is designed to accurately and efficiently predict broiler weight, addressing the time-consuming and labor-intensive nature of manual operations in commercial broiler production. Ultimately, it aims to improve the efficiency of poultry breeding, demonstrating significant potential and broad applicability.

## 1. Introduction

The global consumption of broilers has shown a steady upward trend in recent years, becoming a primary driver of growth in the poultry industry [1,2]. In 2019, global poultry production surpassed that of pork, constituting 35% of the total meat produced worldwide, thereby becoming the most produced meat category. Projections indicate that by 2030, poultry meat is expected to account for 41% of all protein derived from meat [3]. Hence, the assessment of broiler carcass quality has gained significant attention from both producers and consumers, necessitating more precise monitoring methods [4].

In the modern poultry processing industry, the body size and weight of poultry are among the most critical production performance indicators [5]. These metrics not only directly impact slaughter efficiency and economic benefits but are also closely related to feed conversion ratio, breeding selection, and health status assessment. By measuring carcass weight, essential indicators such as slaughter yield and processing uniformity can be comprehensively evaluated, providing crucial data for optimizing slaughterhouse workflows [6,7]. Particularly in large-scale farms, rapidly and accurately obtaining flock weight information is key to achieving intelligent and refined slaughterhouse logistics and management [5,8]. Therefore, for slaughterhouse operators, accurately predicting the carcass weight is essential for developing appropriate grading plans and reducing processing costs [9,10].

Current broiler weight measurement primarily relies on manual weighing and automated platform weighing systems. Manual weighing of broiler carcasses in commercial slaughterhouses is labor-intensive and inefficient, often creating production bottlenecks on high-speed processing lines. Furthermore, manual sampling is susceptible to human error and fails to provide the continuous, real-time data essential for automated grading and precise portion control in modern processing environments [11,12]. However, these weighing systems are primarily designed for live broilers management and are unsuitable for the high-speed environments of automated slaughter lines. In modern processing plants, carcasses are moved rapidly on conveyor hangers, where traditional contact-based weighing is hindered by mechanical vibrations and the risk of cross-contamination. Therefore, there is a critical need for non-contact, vision-based systems capable of individual carcass weight estimation in real-time [13]. With the advancement of artificial intelligence, image processing, and computer vision technologies, researchers have begun to explore more efficient and non-invasive weight estimation methods, with image-based broiler weight prediction systems emerging as a research hotspot. These methods acquire images or videos of broilers and utilize image processing and deep learning techniques to establish regression models for automated chicken weight prediction. This approach eliminates the need for physical intervention, avoiding the errors, labor costs, and negative impacts on animal welfare associated with manual weighing [14].

Guo et al. [15] developed a laying hen weight prediction model using top-down images combined with image processing techniques. The model achieved a coefficient of determination ($R^{2}$) of 0.960 and an average relative error of 4.29%, validating the feasibility of this method for poultry weight prediction. Nyalala et al. [13] further proposed a broiler carcass weight prediction system based on depth images, using an active shape model to segment the carcass into four regions (thighs, breast, wings, and head/neck). By constructing region-specific regression models, the Bayesian neural network model demonstrated optimal performance, with correlation coefficients for carcass and cut weight predictions as high as 0.9981 and 0.9847, respectively, showcasing excellent fitting performance. Furthermore, Adamczak et al. [16] designed an imaging system that combines a 3D scanner with a rotating platform to acquire 3D images of chicken carcasses and extract cross-sectional surface areas to estimate breast meat weight, thus achieving more precise non-contact measurements. Amraei et al. [17] utilized image processing methods to extract characteristics from broilers, employing elliptical fitting to locate chickens and Chan-Vese segmentation to remove heads and tails, and constructed a second-order dynamic regression model for weight prediction. Their model achieved a $R^{2}$ value of 0.98, indicating a high consistency between the predicted and actual measured values.

Despite the significant progress achieved in the aforementioned studies, there remain discernible gaps worth exploring from the perspectives of technical implementation and application promotion. First, at the level of segmentation algorithms, traditional methods such as Active Shape Models, elliptical fitting, and Chan-Vese, while effective under specific conditions, exhibit disparities when compared to deep learning-based semantic segmentation networks (e.g., U-Net) in terms of model representation capability, adaptability to complex morphologies, and degree of automation. Second, concerning data acquisition, although 3D imaging technology provides rich three-dimensional information, its high hardware costs and the complexity of data processing constitute obstacles to its large-scale deployment in fast-paced environments like slaughter production lines.

Therefore, this study aims to explore a more robust and cost-effective solution by proposing an automated broiler carcass weight prediction method that integrates deep learning with regression analysis. Specifically, the objectives are as follows: (1) To achieve high-precision segmentation of broiler carcass images by applying R2U-Net combined with CBAM and SKA attention mechanisms; (2) To extract the pixel area from the segmentation results and construct and optimize a weight prediction regression model based on the relationship between pixel area and actual measured weight. This method is expected to enhance prediction accuracy and automation while reducing hardware requirements.

Furthermore, in the field of carcass image analysis of livestock and poultry carcasses, prior studies have incorporated attention mechanisms into U-Net family architectures or adopted transformer-based segmentation frameworks to improve segmentation accuracy and support downstream applications, such as tissue/part segmentation, body-size measurement, live-animal weight estimation, and carcass defect detection. To more clearly define the positioning of this work and its incremental contribution, we summarize representative related studies in Table 1.

## 2. Materials and Methods

### 2.1. Broiler Chicken Individual Part Weight Collection

Healthy market-age Taihu Yellow chickens (58–60 days old), a premium yellow-feathered broiler breed, were selected as experimental animals. All samples were obtained from standardized commercial farms operated by Lihua Livestock and Poultry Co., Ltd. (Changzhou, China). A total of 301 broiler carcasses were collected for subsequent analysis to ensure adequate statistical power and representativeness. Prior to slaughter, all broilers were subjected to a 12 h feed withdrawal with free access to water to empty the gastrointestinal tract. Broilers were then stunned using a carbon dioxide (CO_2_) gas mixture by placing them in a CO_2_ stunning chamber with a gradually increasing CO_2_ concentration, resulting in rapid loss of consciousness. Immediately after stunning, broilers were euthanized by exsanguination via severing the carotid artery. Following bleeding, carcasses were scalded in a 60 °C water bath for approximately 90 s, mechanically defeathered using a drum plucker, and subsequently rinsed. During image acquisition and subsequent sampling/analysis, carcasses were maintained intact with viscera retained. All phenotypic measurements were performed after image acquisition and prior to carcass cooling in a pre-chilling tank. To ensure consistency and accuracy, all measurements were conducted by the same trained personnel.

The carcass weight (CW) was determined using a Baijie I-2000 stainless steel kitchen scale (Zhejiang Junkai Shun Industry and Trade Co., Ltd., Yongkang, China) and a Ruijian Hengqi JKS-5605 electronic scale (Zhejiang Junkai Shun Industry and Trade Co., Ltd., Yongkang, China) (accuracy 1 g). Morphometric measurements were taken with a 2-m standardized soft ruler (minimum increment 1 mm) and a Deli DL90150 industrial grade caliper (Deli, Ningbo, China) (accuracy 0.02 mm). Before measurement, each carcass was suspended vertically for 10 min to allow residual surface water to drain naturally, thus eliminating water interference with the weighing results. Subsequently, each carcass was placed centrally in the dry pan of the electronic scale. Once the reading stabilized, the value was recorded. To improve data reliability, each carcass was weighed three times, with its placement posture altered for each measurement. The arithmetic mean of these measurements was taken as the final CW. Immediately after weighing, each carcass was fitted with a unique leg band number for sample traceability and data matching.

Finally, the carcasses were meticulously dissected to remove all internal organs. This procedure was performed to separate the carcass into primary cuts of commercial and research significance, including entire wings, breast meat, whole legs, feet, and the head and neck region. All resultant paired parts, such as the left and right wings, legs, and breast muscles, were individually weighed using an electronic scale. The data were recorded for subsequent analysis of meat yield performance and body symmetry.

### 2.2. Image Acquisition

#### 2.2.1. Image Acquisition System and Environment

**Figure 1:**
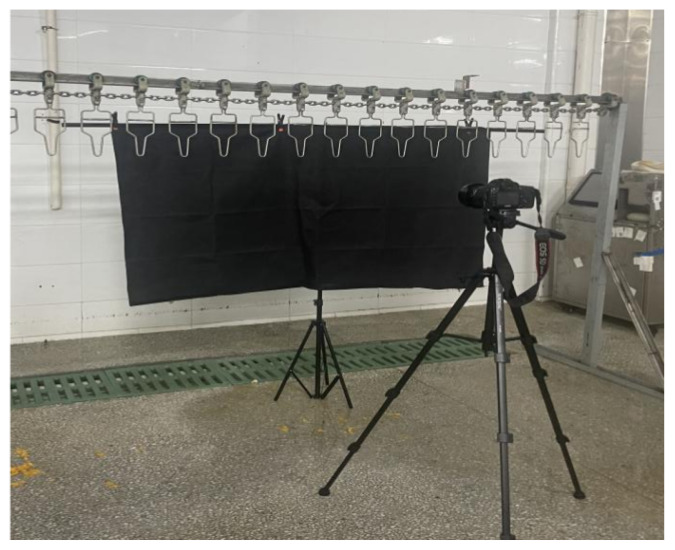
Image acquisition field map.

The image acquisition was conducted on the production line of Jiangsu Lihua Food Co., Ltd. (Changzhou, China). The collection point was established after the plucking process and before the evisceration and cooling stages, ensuring the integrity of the carcass surface and preventing any effects from subsequent processing. To simulate a realistic industrial production scenario and standardize image acquisition, an image acquisition platform, as illustrated in Figure 1, was created. This platform mimicked a conveyor belt suspension mode, with chicken carcasses vertically suspended for photography. The acquisition environment was located within the production workshop, where ambient light was both sufficient and stable, eliminating the need for additional artificial lighting. To simplify the image background and emphasize the features of the carcass, a 1.6 m × 1.0 m black background cloth (LATZZ, Shenzhen, China) was positioned behind the conveyor line hangers and secured with a LATZZ 2 m × 2 m T-shaped photographic background stand (LATZZ, Shenzhen, China). The image acquisition device used was a Canon EOS 5D Mark III digital SLR camera (Canon, Tokyo, Japan) equipped with a 35 mm prime lens. The camera was mounted on a YUNTENG VT-888 tripod (YUNTENG, Zhongshan, China) to ensure stability and consistency during the shooting process.

#### 2.2.2. Image Acquisition Protocol

During the image acquisition process, each yellow-feathered broiler carcass was removed from the production line and re-suspended on a fixed photographic hanger. This method ensured that the carcass’s center of gravity remained stable and was directly aligned with the camera lens. The vertical distance between the camera and the carcass was fixed at 120 cm, a measurement selected to guarantee that the entire carcass, from the neck to the tip of the leg, was captured within the frame.

To ensure consistent exposure and depth of field across all images, the camera settings were configured to manual mode and fixed as follows: a focal length of 35 mm, an aperture of f/3.5, and an exposure time of 1/30 s. The ISO was automatically adjusted based on the ambient light conditions.

**Figure 2:**
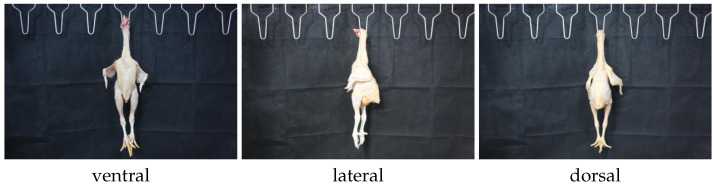
Three standardized perspectives.

Each chicken carcass was photographed from three standardized perspectives: ventral, dorsal, and lateral views. The specific definitions for these orientations are illustrated in Figure 2. To minimize random errors and ensure data quality, three images were captured for each view. Consequently, a total of 3 × 3 = 9 high-resolution images were obtained per chicken carcass, resulting in 301 × 9 = 2709 raw images for the entire experiment.

#### 2.2.3. Image Processing

Semantic segmentation annotations were created using Labelme for a dataset of 2709 broiler chicken images, in which the complete broiler region and key anatomical parts were meticulously delineated. The resulting annotations were then converted to a format compatible with deep learning models, such as COCO [25] or YOLO [26]. To facilitate model training and evaluation, the dataset was partitioned into training, validation, and testing subsets using an 8:1:1 split at the carcass level. Because each carcass corresponds to multiple images (multi-view and repeated captures), a carcass-wise (subject-independent) split was employed to prevent data leakage: all images from the same carcass were assigned exclusively to a single subset and were not shared across subsets. The training subset was used to learn model parameters, the validation subset to optimize hyperparameters and monitor generalization, and the held-out test subset to provide a final unbiased evaluation of segmentation accuracy. The performance of our model was quantified by comparing the predicted segmentation masks against the ground truth labels on the test set, using standard evaluation metrics.

### 2.3. Deep Learning Models

U-Net [27], proposed by Olaf Ronneberger et al. in 2015, is a foundational image segmentation algorithm specifically designed for medical imaging tasks. Its core concept is to achieve high-precision, pixel-level segmentation by utilizing a symmetric encoder-decoder architecture with skip connections. However, the U-Net decoder relies on features from a single encoder level for upsampling, lacking a mechanism for the dynamic fusion of multi-scale features. To address this limitation, U-Net++ [28] introduces dense connections along the skip pathways. This enhancement facilitates a more comprehensive integration of multi-scale features and reduces the semantic gap between the encoder and decoder feature maps. Furthermore, the standard U-Net architecture lacks mechanisms to ensure efficient gradient flow and feature reuse as the network deepens, making it susceptible to vanishing gradients and network degradation. The Residual U-Net [29] addresses this issue by incorporating residual blocks, which introduce identity mapping through short connections within each encoder and decoder unit. The Residual U-Net has limitations in its ability to capture long-range dependencies and refine features through multiple iterations. The R2U-Net (Recurrent Residual U-Net) [30] incorporates recurrent convolutional modules within its residual units. This integration allows for multiple recursive feature extractions, thereby enhancing the network’s capacity to capture complex structural details.

**Figure 3:**
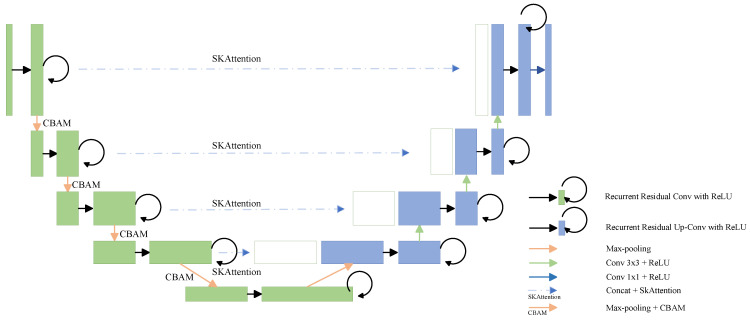
Structure diagram of AR2U-AttnNet.

In research focused on the image segmentation of broiler carcasses for dynamic weight detection, achieving high model accuracy and robustness is essential. Industrial processing lines often present significant challenges for segmentation tasks, including complex background interference, variable lighting conditions, and variations in object scale and posture. Our proposed model is built upon the R2U-Net architecture, which is well-known for its exceptional ability in feature reuse and gradient propagation, thanks to its inherent recurrent residual convolutional units. To further enhance the model’s feature representation and discrimination capabilities in complex scenarios, the Convolutional Block Attention Module (CBAM) [31] and the Selective Kernel Attention Module (SKAttention) [32] are integrated. The resulting architecture is termed AR2U-AttnNet (Attentive Recurrent U-Net with Dual Attention Modules). The model structure is shown in Figure 3. This integration aims to synergistically improve the network’s perception and focus across three dimensions: feature channels, spatial dimensions, and receptive field scales.

### 2.4. CBAM Attention Mechanism

**Figure 4:**
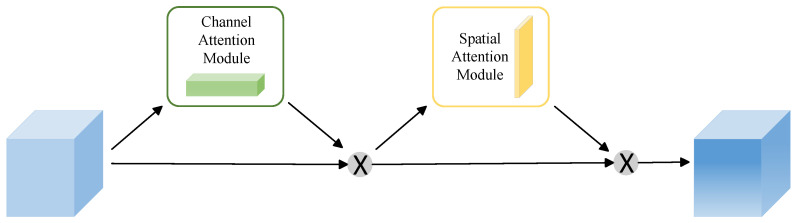
Structure diagram of CBAM.

Although the residual and recurrent mechanisms in R2U-Net enhance feature accumulation and refinement, they do not incorporate an explicit, dynamic attention mechanism to emphasize “what” is most important. In practical scenarios, such as uneven illumination, varying postures of broiler chickens, or partial occlusion, the model may struggle to automatically identify and prioritize the key pixels and features that are most indicative of broiler locations and boundaries. This limitation can lead to less robust segmentation performance. To address these challenges, the introduction of the Convolutional Block Attention Module (CBAM) is particularly effective. The structure of CBAM is shown in Figure 4.

**Figure 5:**
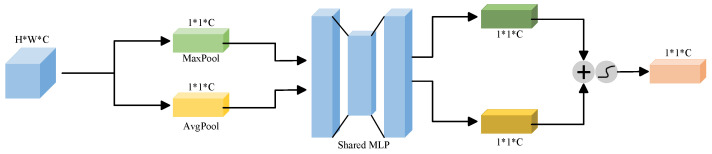
Structure diagram of CAM.

**Figure 6:**
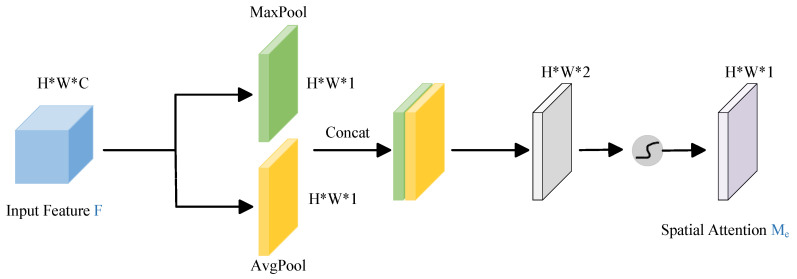
Structure diagram of SAM.

The CBAM attention mechanism is divided into two parts: spatial attention module and channel attention module. The structure of each part is shown in Figure 5 and Figure 6. The Channel Attention Module (CAM) in the CBAM first aggregates the spatial information of each channel by applying both average pooling and max pooling operations in parallel along the spatial dimension [33]. The resulting pooled features are then passed through a shared Multi-Layer Perceptron (MLP) to learn and generate channel-wise attention weights. Finally, these weights are applied to the original feature map through element-wise multiplication, enhancing salient feature channels while suppressing less informative ones. The calculation is as follows:

(1) $$\begin{array}{cc} M_{c}\left(F\right) & =\sigma (MLP(AvgPool(F))+MLP(MaxPool(F)))\\ \; & =\sigma (W_{1}\left(W_{0}\left(F_{avg}^{c}\right)\right)+W_{1}(W_{0}(F_{max}^{c}))) \end{array}$$

where $M_{c}\left(F\right)$ is the channel attention output, σ is the Sigmoid activation function, MLP is the Multilayer Perceptron, AvgPool(F) is the global average pooling, MaxPool(F) is the global maximum pooling, $W_{0}$ and $W_{1}$ are the weight matrix of the MLP, $F_{avg}^{c}$ is the global average pooling feature, $F_{max}^{c}$ is the global maximum pooling feature.

The Spatial Attention Module (SAM) in the CBAM primarily focuses on spatial information. It begins by applying global average pooling and global max pooling to the input feature map. The outputs of these two pooling operations are then concatenated. This concatenated feature map is passed through a convolutional layer to generate a spatial attention map, which is subsequently multiplied with the original feature map to emphasize important spatial regions.The calculation is as follows:

(2) $$\begin{array}{cc} M_{s}\left(F\right) & =\sigma (f^{7\times 7}\left(|AvgPool\left(F\right);MaxPool\left(F\right)|)\right)\\ \; & =\sigma (f^{7\times 7}\left(\left[F_{avg}^{c};F_{max}^{c}\right]\right)) \end{array}$$

where $M_{s}\left(F\right)$ is the spatial attention output, $f^{7\times 7}$ represents a 7 × 7 convolution operation.

Adding the CBAM to a convolutional neural network significantly enhances the model’s capacity to represent image features. This is accomplished by concurrently emphasizing important information in both the channel and spatial dimensions, allowing the network to adaptively amplify meaningful features while suppressing less significant ones. As a result, the overall performance of the model is improved. Therefore, this plays an essential role in accurately segmenting the target outline from a cluttered background, thereby ensuring the integrity and precision of the segmentation.

### 2.5. SKAttention Mechanism

**Figure 7:**
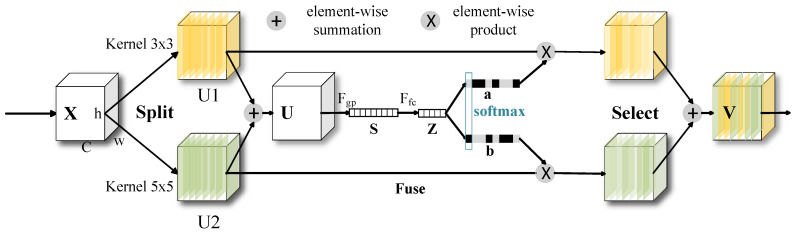
Selective kernel attention mechanism module.

Meanwhile, SKAttention module effectively addresses the limitations of fixed receptive fields in traditional Convolutional Neural Networks (CNNs), allowing models to dynamically adapt to variations in target scale. In the context of broiler dynamic detection, the apparent size of targets in images varies significantly due to differences in camera distance, carcass size, and placement posture. Fixed convolutional kernel sizes struggle to simultaneously capture both global structural information for large-scale targets and local details for small-scale targets. SKAttention overcomes this challenge through a “Split-Fuse-Select” strategy.The module is illustrated in Figure 7.

Specifically, the input feature map *X* is processed in parallel by multiple branches that utilize different kernel sizes (e.g., 3 × 3, 5 × 5). This parallel operation enables the extraction of multi-scale feature information, resulting in several feature maps ($U_{1}$,$U_{2}$), each corresponding to a distinct receptive field. These feature maps are subsequently fused to obtain a new feature map U, as illustrated in Formula (3):

(3) $$\begin{array}{c} U=U_{1}⊕U_{2} \end{array}$$

where ⊕ represents element-wise summation.

Then, we embed global information through global average pooling. Assuming the given input feature tensor is $U=[u_{1},u_{2},u_{3},\ldots ,u_{C}$, the output $F_{c}$ associated with channel *c* of the global pooling operation is shown in Formula (4). Futher, Formula (5) shows a feature *z* is obtained by further processing through two fully connected layers.

(4) $$\begin{array}{c} s_{c}=F_{gp}\left(U_{c}\right)=\frac{1}{H\times W}\sum_{i=1}^{H}\sum_{j=1}^{W}U_{c}\left(i,j\right). \end{array}$$

where $U_{c}\left(i,j\right)$ is a component of input *U*.

(5) $$\begin{array}{c} z=F_{fc}\left(s\right)=\delta \left(B\left(\mathrm{Ws}\right)\right) \end{array}$$

where δ is the ReLU function, B is the Batch Normalization.

Finally, the attention weights are calculated using Softmax, and the final output V is obtained through weighted fusion, as illustrated in Formula (6):

a,b=Softmax(z)

where *a* and *b* correspond to the attention weights of the two branches, and a+b=1.

(6) $$\begin{array}{cc} V & =aU_{1}+bU_{2} \end{array}$$

Note that this formula is only applicable to two branches, further derivation is needed for multi-branch formulas.

This mechanism enables each unit of the network to adaptively adjust its effective receptive field size based on the characteristics of the current input. When processing a whole chicken carcass that occupies a large portion of the image, the network can prioritize activating branches with larger receptive fields to capture global contour information. Conversely, when fine local details—such as wing tips or partially occluded edges—need to be processed, the network can concentrate on branches with smaller receptive fields to capture intricate features. By incorporating the SKAttention module into the encoder path of R2U-Net, we equip the model with an intrinsic multi-scale analysis capability during the initial stages of feature extraction. This significantly enhances its robustness to scale variations, ensuring consistent and highly accurate segmentation results across various conditions.

### 2.6. Regression Models

**Figure 8:**
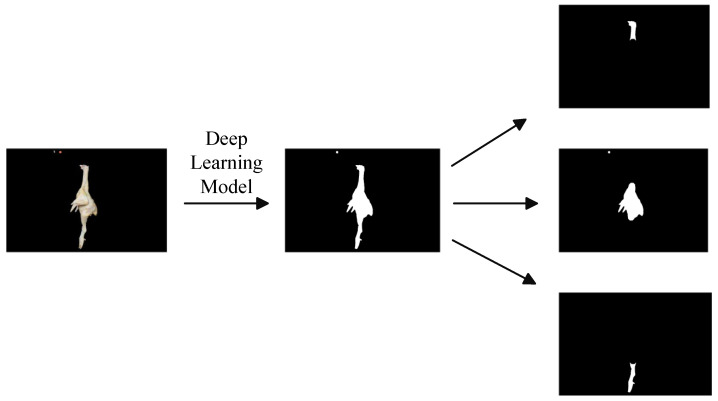
The segmentation results obtained from the original images by the image segmentation model are further processed using morphological operations to generate segmentation maps of the three specific parts.

After obtaining the segmented images from the model, a series of morphological operations were performed on the samples using MATLAB R2021b. First, a structuring element was defined, and a closing operation—comprising dilation followed by erosion—was applied to fill any holes present in the images. Next, an opening operation, which involves erosion followed by dilation, was conducted to eliminate minor noise. This two-step process resulted in morphologically refined images of the various parts of the broiler chicken.The results of each segmentation are shown in Figure 8. Finally, the pixel values for each part were quantified by referencing a calibration object within the image. All acquired data were systematically recorded in an Excel spreadsheet.

In the regression stage, the pixel area of the segmented mask for each anatomical part is adopted as the primary input feature to represent the image-scale information of the carcass and its components. Under acquisition protocol, the camera-to-carcass distance and imaging viewpoints were kept relatively consistent, and the pixel measurements were further calibrated using a reference object within the image; therefore, the area feature is both stable and interpretable. In contrast, boundary-derived shape descriptors (e.g., perimeter, aspect ratio, and convex hull area) are more sensitive to segmentation boundary errors and occlusions. In lateral-view images, overlap between the legs and trunk may blur part boundaries, making such descriptors more prone to noise and potentially reducing the robustness of the regression model. Consequently, the area of the pixels is used as the baseline feature in this study, and the incorporation of additional shape descriptors will be explored in future work.

To analyze and illustrate the relationship between pixel values and body weight, a robust statistical technique known as regression modeling was employed. The regression model used in this research is as follows:

1. Multilayer Perceptron(MLP);
2. Support Vector Regression(SVR(RBF));
3. Bayesian Regression;
4. Light Gradient Boosting Machine(LightGBM);
5. Categorical Boosting(CatBoost).

The theoretical foundations and mathematical structures of these regression models are available in the cited references [34,35,36,37,38]. The regression models were developed using the same train/validation/test split as described earlier. The training set was used for model fitting, the validation set for model selection and hyperparameter setting, and the test set was reserved for final unbiased evaluation. We did not perform k-fold cross-validation in this study and will consider it in future work to further assess robustness.

### 2.7. Experimental Platform

This experiment was conducted using the Python programming language. The software environment comprised Windows 11, PyTorch 2.2.2, Python 3.10.14, and CUDA 12.1 for consistency and objectivity. The hardware environment included an NVIDIA RTX 4090 GPU with 24 GB of VRAM and an Intel Core i7-13700K CPU. The input image size was configured to 640 × 640 pixels, with a batch size of 8. The model was trained for 200 epochs, starting with an initial learning rate of 0.001.

### 2.8. Evaluation Metrics

To objectively and quantitatively evaluate the effectiveness of our proposed broiler image segmentation model, we employed three key evaluation metrics widely used in semantic segmentation: Mean Intersection over Union (mIoU), Dice coefficient, and F1-Score.

$$\mathrm{IoU}=\frac{\left|A\cap B\right|}{\left|A\cup B\right|}$$

where *A* represents the predicted segmentation result set of the model, while *B* represents the set of manually annotated true regions (Ground Truth).

mIoU is obtained by calculating the IoU values of all categories in the dataset and taking their average. The formula is as follows:

(7) $$\begin{array}{c} \mathrm{mIoU}=\frac{1}{n}\sum_{i=1}^{n}{\mathrm{IoU}}_{i} \end{array}$$

The Dice coefficient functions similarly to IoU; it is also used to measure the similarity between two samples, particularly excelling in evaluating the agreement of segmentation boundaries. The formula is as follows:

(8) $$\begin{array}{c} \mathrm{Dice}=\frac{2\times |A\cap B|}{\left|A|+|B\right|} \end{array}$$

The F1 score is the harmonic mean of precision and recall, which effectively balances the relationship between the two, providing a more comprehensive reflection of the model’s performance. The relevant calculation formulas are as follows:

$$\mathrm{Precision}=\frac{\mathrm{TP}}{\mathrm{TP}+\mathrm{FP}}$$

$$\mathrm{Recall}=\frac{\mathrm{TP}}{\mathrm{TP}+\mathrm{FN}}$$

(9) $$\begin{array}{c} F\mathrm{1-Score}=2\times \frac{\mathrm{Precision}\times \mathrm{Recall}}{\mathrm{Precision}+\mathrm{Recall}} \end{array}$$

where TP refers to the number of chicken pixels that are correctly segmented; FP refers to the number of background pixels that are incorrectly segmented as chicken; while FN indicates the number of chicken pixels that the model has missed.

## 3. Results

In this section, we will focus on the performance results of the proposed image segmentation and regression models.

### 3.1. Segmentation Result

To verify the effectiveness of the proposed AR2U-AttnNet model, comparative and ablation experiments were conducted. Several advanced U-Net variants and other segmentation models were evaluated, including Attention-UNet [39], UNet++, SwinUNet [40], TransUNet [41], and DeepLabV3+ [42]. The experimental results are shown in Table 2.

From the Table 2, the AR2U-AttnNet model consistently outperforms all other benchmark models in every metric evaluated. It achieves the highest mIoU at 90.45%, along with a Dice coefficient of 95.18%, precision of 95.73%, recall of 94.64%, and an F1 score of 95.18%. These results indicate that the proposed model has a significant advantage in accurately segmenting targets while maintaining a strong balance between precision and recall.

SwinUNet emerged as the best-performing model among all baseline architectures, achieving a mean mIoU of 88.89% and a Dice coefficient of 94.13%. This indicates the robust feature extraction and context modeling capabilities of Transformer-based architectures in this segmentation task. However, our model outperformed SwinUNet by 1.56 percentage points in mIoU and 1.05 percentage points in the Dice coefficient, demonstrating superior segmentation accuracy.

TransUNet, a model that integrates Transformer and U-Net architectures, achieved commendable results with a mIoU of 86.64%. However, its performance significantly lagged behind both SwinUNet and our proposed model.

The classic DeepLabV3+ model achieved a mIoU of 83.51%. However, its performance did not match that of the Transformer-based models and was significantly inferior to our proposed model.

UNet++ (mIoU 82.25%) and Attention-UNet (mIoU 81.08%) have made some improvements over the traditional U-Net, but there is still a significant gap in their ability to handle complex scenes compared to our model and SwinUNet.

**Figure 9:**
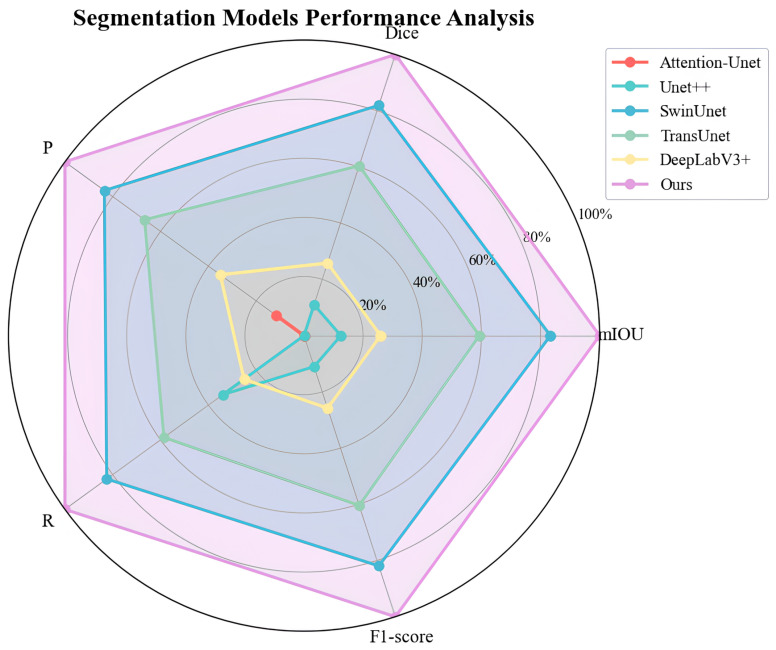
Radar chart of segmentation model performance. The radar chart displays model performance through the shape and area of a polygon. The farther the data points are from the center, the better the performance of that metric. A larger polygon area indicates stronger overall model performance.

Figure 9 more intuitively demonstrates the superiority of the AR2U-AttnNet model. Data were normalized to evaluate the performance of the model. Normalization was performed using the Min-Max scaling formula as follows:

(10) $$x^{\prime }=\frac{x-x_{\min}}{x_{\max}-x_{\min}}$$

where *x* is the original value, $x_{\min}$ and $x_{\max}$ are the minimum and maximum of the dataset, respectively, and $x^{\prime }$ denotes the normalized value.

Ablation experiments can be used to verify the effectiveness of each component in the model. Beyond the established performance metrics of mIoU, Dice, and F1-score, we incorporated GFLOPs to quantify the computational expense and the Number of Parameters to gauge the model’s complexity.The experimental results are shown in Table 3.

Compared to the baseline model (mIoU: 88.57%, Dice coefficient: 93.89%, F1-score: 93.89%), the introduction of either CBAM or SKAttention nhances the model’s performance. The integration of CBAM alone results in a 0.64% increase in MIoU and a 0.43% increase in both the Dice coefficient and F1-score. The exclusive introduction of SKAttention leads to a 1.21% improvement in mIoU and a 0.71% enhancement in both the Dice coefficient and F1-score. Optimal performance is achieved when both CBAM and SKAttention are incorporated simultaneously, yielding an mIoU of 90.45% and Dice and F1-scores of 95.18%. This indicates that the two attention mechanisms can complement each other, achieving a synergistic effect that further enhances the model’s segmentation capabilities.

Adding attention mechanisms slightly increases the computational complexity of the model and the number of parameters. The baseline model has 82.27 GFLOPs and 43.86 million parameters. When two attention mechanisms are introduced, the GFLOPs increase by 1.3%, and the number of parameters increases by 0.89% compared to the baseline. Although there is a minor increase in computational complexity and parameters, this is outweighed by a significant improvement in performance.

**Figure 10:**
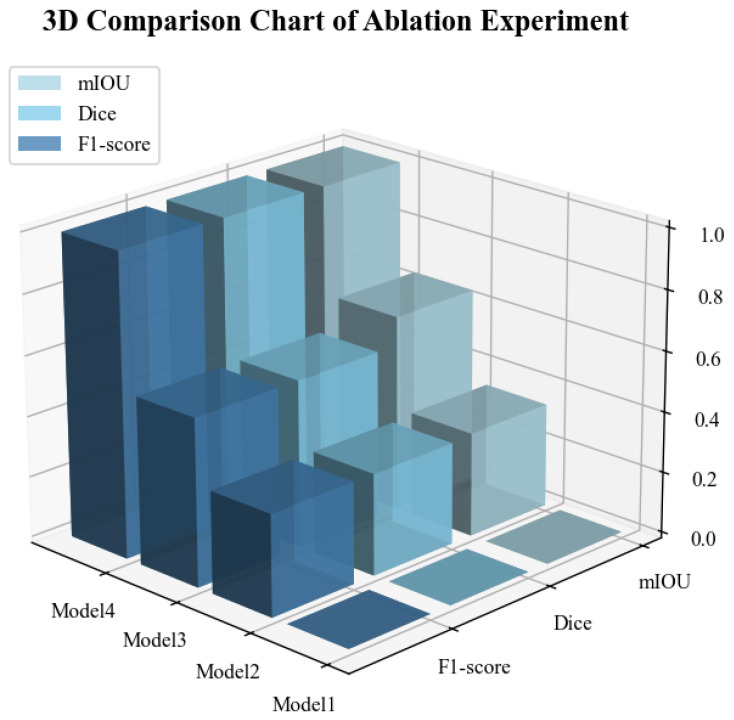
3D Comparison Chart (Normalized). The Z-axis (vertical height) represents the scores of each model on specific metrics, with values ranging from 0.0 to 1.0. A higher score indicates better performance. Model 1 represents the baseline, Model 2 represents the one that only added CBAM, Model 3 represents the one that only added SKAttention, and Model 4 represents AR2U-AttnNet.

Figure 10 intuitively displays the performance of each module. Here, Formula (10) is also utilized.

### 3.2. Regression Result

Data on various components of broiler chickens were collected and organized into four modules, designated as Mod1 through Mod4.

Mod1 focuses on the overall surface area of the broiler from the ventral, lateral, and dorsal perspectives. Mod2 addresses the carcass surface area from the same perspectives. Mod3 pertains to the head surface area, while Mod4 examines the leg surface area, all from ventral, lateral, and dorsal viewpoints.

To model the relationship between surface area and body weight, a predictive model of total broiler weight was developed using the three measurements of overall surface area (ventral, lateral, and dorsal) as input features. This methodology was similarly applied to the other components, utilizing the area measurements for the carcass, head, and legs to predict their respective weights.

We use $R^{2}$ and RMSE to measure the performance of the regression model. The coefficient of determination ($R^{2}$) assesses the extent to which the variability in the dependent variable can be accounted for by the model, with values spanning from 0 to 1. A higher $R^{2}$ value, approaching 1, suggests a stronger fit of the model to the data. Conversely, the Root Mean Squared Error (RMSE) provides a measure of the average difference between the model’s predicted outputs and the observed true values. A lower RMSE value is indicative of superior predictive precision.

**Figure 11:**
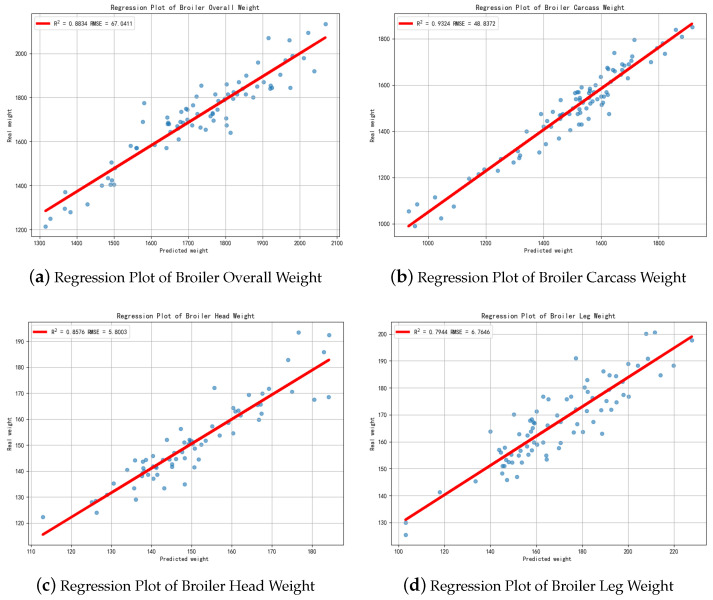
Regression results of the weight of various parts of broiler chickens.Each blue dot denotes one broiler sample, illustrating the relationship between the predicted weight and the corresponding ground-truth measurement.

The experimental results are shown in Table 4. The experimental results are shown in Table 4, while the 95% confidence intervals (CI) are reported in Table 5 and Table 6. The best results for each Mod are shown in Figure 11.

In Mod1, the Bayesian model achieved the highest $R^{2}$ value of 0.8834 and a low RMSE of 67.04g. For Mod2, the CatBoost model demonstrated superior performance with the highest $R^{2}$ of 0.9324 and the lowest RMSE of 48.84g. Similarly, for Mod3, CatBoost was the top-performing model, had the best regression results of 0.8576 $R^{2}$ at an RMSE of 5.80 g. In the case of Mod4, the MLP model yielded the best results with an $R^{2}$ of 0.7944 and an RMSE of 6.76 g.

**Figure 12:**
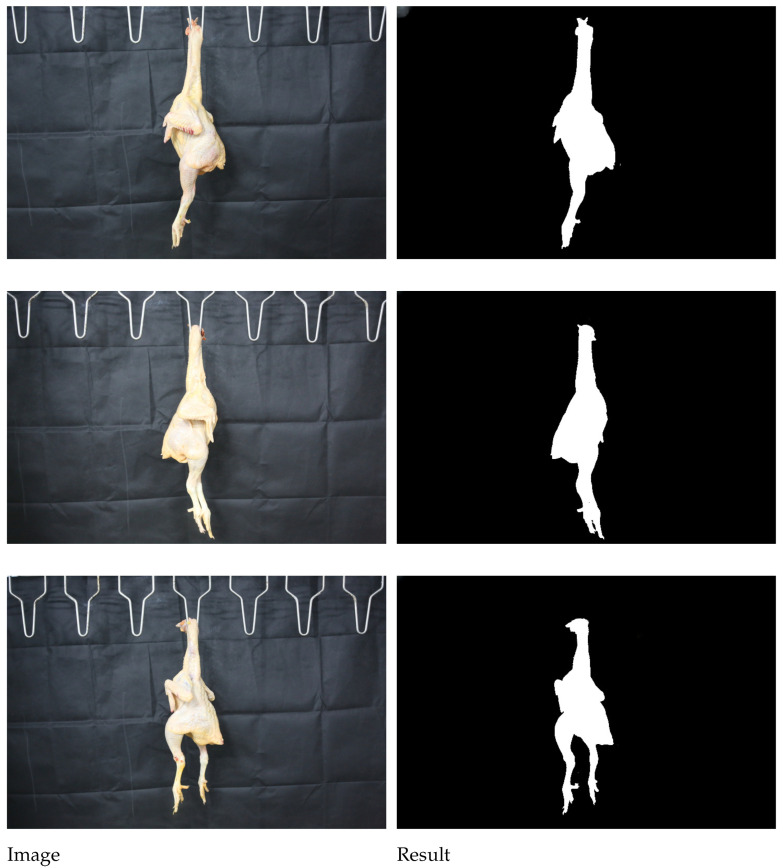
Model segmentation results.

Figure 12 illustrates the segmentation result produced by the AR2U-AttnNet model.

The segmentation results presented in Figure 12 demonstrate that the model can efficiently and accurately extract the complete silhouette of the chicken carcass from the background. The model successfully identifies the metal hook used for suspension as part of the background and excludes it from the segmentation mask. This performance strongly indicates that the model has learned not just simple color or edge information, but intrinsic high-level biomorphological features of the chicken carcass.

However, the validation environment in the current study is relatively idealized. The actual environment of a slaughterhouse production line is far more complex than the uniform background used in our experiments, and the generalizability of the model when faced with samples of varying breeds, sizes, and unusual postures remains to be verified. During the course of the investigation, instances of incomplete segmentation were observed, particularly when processing the chicken feet. Furthermore, observations from side-view images reveal that the thigh is connected to the torso with significant visual overlap and an indistinct boundary line. This presents a considerable challenge for computer vision processing, making precise segmentation difficult and leading to inaccuracies in the prediction results. Therefore, acquiring accurate predictions for the leg portions of the broiler may require the acquisition of three-dimensional images.

**Figure 13:**
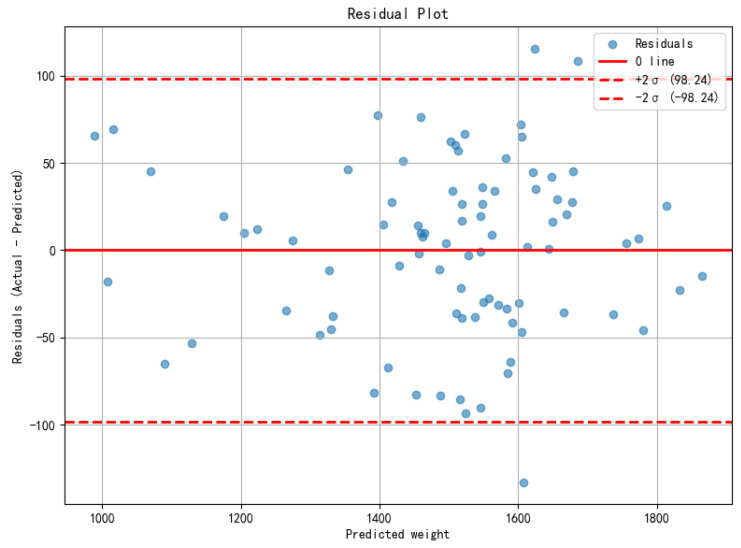
The x-axis shows predicted values and the y-axis shows residuals (Actual—Predicted). The solid red line marks zero residual, and the dashed red lines denote ±2σ (σ: residual SD) to highlight outliers and error dispersion.

Furthermore, to further analyze the error distribution and stability, the residual scatter plot of the best-performing model in Mod2 on the test set is shown in Figure 13. The best model was selected based on overall performance in terms of $R^{2}$ and RMSE.

## 4. Discussion

### 4.1. Comparison with End-to-End Approaches

We adopted a two-stage segmentation–regression framework, with an emphasis on interpretability and engineering controllability. The segmentation stage explicitly localizes the carcass and key anatomical parts, restricting the regression input to anatomically meaningful regions, which facilitates quality inspection and error tracing while reducing the model’s reliance on irrelevant information under complex backgrounds. In contrast, end-to-end approaches directly regress weight from raw images, offering a simpler pipeline and, in principle, the ability to exploit appearance cues such as texture and morphology. However, in processing-line scenarios with illumination changes, pose variations, and background interference, end-to-end models are more prone to learning spurious correlations unrelated to weight and typically require substantially larger datasets to achieve robust generalization. Based on these considerations, we use the two-stage framework as our baseline and plan to incorporate end-to-end baselines and joint-learning strategies in future work.

### 4.2. Limitations and Future Work

By pivoting the focus of the surveillance from live poultry to carcasses, this study effectively mitigates the volumetric interference of feathers and movement-induced postural variations, significantly improving the robustness of weight prediction. Despite these technical advancements, structural barriers to large-scale industrial deployment persist; specifically, the current framework’s reliance on semi-idealized backgrounds and stable illumination may not fully accommodate the complex environments of commercial slaughterhouses, characterized by water mist and metallic reflections. Furthermore, the inherent constraints of 2D pixel-area-based regression lead to significant anatomical overlap between the thighs and torso in lateral views. This “occlusion effect” restricts the predictive precision of localized modules, such as Mod4. Additionally, as the current dataset is limited to yellow-feathered broilers within a specific age range, the model’s generalizability across diverse breeds and processing states requires further empirical validation.

To transcend these limitations, future research will prioritize the development of dual-modality data fusion schemes. By synergistically integrating RGB textural information with structural depth features, the system can leverage cross-modal complementarity to enhance semantic comprehension under heterogeneous industrial conditions. This multimodal perception architecture offers a fundamental solution to the 2D masking effect, enabling high-precision, orientation-agnostic weight estimation through the extraction of volumetric biomorphological features. Coupled with transfer learning on multi-breed datasets and model optimization for edge-device deployment, this roadmap provides a robust path toward real-time, non-contact monitoring and automated grading in high-speed slaughterhouse operations.

## 5. Conclusions

To address the inefficiencies and inaccuracies of traditional methods for determining the weight of carcasses, this study constructed and validated a novel deep learning-based model for the detection of dynamic chicken carcass weight. This model is composed of an innovative segmentation network, AR2U-AttNet, and an efficient regression model. The experimental results substantiate the effectiveness of the proposed method. Our segmentation model, AR2U-AttNet, demonstrated exceptional performance in accurately identifying and localizing chicken carcasses from complex backgrounds, achieving an mIoU of 90.45%, and a Dice coefficient and F1-score of 95.18%. Building upon this precise segmentation, the subsequent regression model efficiently learned the mapping relationship between image features and actual weight. This was evidenced by $R^{2}$ values of 0.9324, indicating high reliability and accuracy in weight prediction. This research successfully develops a high-precision automated solution that significantly outperforms traditional methods in accuracy, efficiency, and robustness, demonstrating considerable potential for future applications.

## Figures and Tables

**Table 1:** Representative attention-based studies in livestock and poultry imaging.

Ref.	Year	Object	Model	Difference
Cao et al. [18]	2023	Sheep loin CT	Attention-UNet	CT sheep; ours RGB carcass + weight
Shi et al. [19]	2023	Cattle(side view)	U-Net variant (A-Unet)	Body-size only; ours weight monitoring
Tran et al. [20]	2024	Poultry carcass	Transformer end-to-end	Defect focus; ours weight prediction
Xu et al. [21]	2024	Cattle	SE attention + regression	SE+BP; ours CBAM + SK + regression
Li et al. [22]	2025	Laying hens	Transformer segmentation(cross-scale attention)	Live hens; ours carcasses
Li et al. [23]	2025	Pig/Horse	U-Net + transformer	Generic livestock; ours R2U + dual-attn
Chen et al. [24]	2025	Chicken parts	Lightweight + SENet(attention)	Parts only; ours whole-carcass + weight

**Table 2:** Comparison of AR2U-AttnNet with various image segmentation models.

Model	mIoU ^2^	Dice ^3^	P ^4^	R ^5^	F1-Score ^6^
Attention-UNet	81.08	89.37	90.05	88.70	89.37
UNet++	82.25	90.01	89.31	90.72	90.01
SwinUNet	88.89	94.13	94.67	93.60	94.13
TransUNet	86.64	92.88	93.59	92.18	92.88
DeepLabV3+	83.51	90.87	91.56	90.19	90.87
Ours ^1^	90.45	95.18	95.73	94.64	95.18

^1^ Ours represents the model AR2U-AttnNet that we proposed, ^2^ mIoU represents Mean Intersection over Union, ^3^ Dice represents Dice coefficient, ^4^ P represents Precision, ^5^ R represents Recall, ^6^ F1 score represents the harmonic mean of precision and recall.

**Table 3:** Comparison of ablation test results for AR2U-AttnNet.

Model	CBAM	SKAttention	mIOU	Dice	F1-Score	GFLOPs (G)	Parameters (M)
R2U-Net	**✗** ^1^	**✗**	88.57	93.89	93.89	82.27	43.86
(Baseline)	**✓** ^2^	**✗**	89.21	94.32	94.32	82.45	43.92
	**✗**	**✓**	89.78	94.60	94.60	83.12	44.19
	**✓**	**✓**	90.45	95.18	95.18	83.31	44.25

^1^**✗** indicates that the module is not included, ^2^ **✓** indicates that the module is included.

**Table 4:** Regression models performance comparison.

	*Mod1*		*Mod2*		*Mod3*		*Mod4*	
Regression Models	$R^{2}$	RMSE	$R^{2}$	RMSE	$R^{2}$	RMSE	$R^{2}$	RMSE
MLP	0.8794	68.18	0.9147	54.83	0.7772	7.26	0.7944	6.76
RBF-SVR	0.8241	82.34	0.8509	72.51	0.7516	7.66	0.6307	9.06
Bayes	0.8834	67.04	0.8881	62.81	0.5675	10.10	0.6734	8.74
LGBM	0.8379	79.03	0.8625	69.64	0.7639	7.47	0.6259	9.12
CatBoost	0.8651	69.55	0.9324	48.84	0.8576	5.80	0.7376	7.64

**Table 5:** $R^{2}$ (95% CI) on the test set for different regression models.

Models	*Mod1*	*Mod2*	*Mod3*	*Mod4*
MLP	[0.8022, 0.9236]	[0.8541, 0.9487]	[0.6792, 0.8554]	[0.6700, 0.8602]
RBF-SVR	[0.7163, 0.8845]	[0.7877, 0.8919]	[0.6209, 0.8426]	[0.4749, 0.7249]
Bayes	[0.8048, 0.9256]	[0.8323, 0.9232]	[0.2421, 0.7566]	[0.4973, 0.7361]
LGBM	[0.7358, 0.8923]	[0.7896, 0.9036]	[0.6264, 0.8613]	[0.4880, 0.7255]
CatBoost	[0.8163, 0.9286]	[0.8908, 0.9542]	[0.7877, 0.9081]	[0.6075, 0.8140]

**Table 6:** RMSE (95% CI) on the test set for different regression models.

Models	*Mod1*	*Mod2*	*Mod3*	*Mod4*
MLP	[55.43, 79.93]	[44.86, 65.60]	[5.83, 8.70]	[5.72, 7.80]
RBF-SVR	[68.98, 94.31]	[62.32, 82.89]	[6.10, 9.16]	[7.61, 10.33]
Bayes	[55.73, 78.18]	[52.59, 72.47]	[7.35, 13.55]	[7.08, 10.01]
LGBM	[67.59, 90.38]	[61.79, 77.45]	[5.67, 9.27]	[7.57, 10.69]
CatBoost	[55.11, 78.59]	[42.54, 55.40]	[4.60, 7.02]	[6.37, 8.83]

## Data Availability

As this is a proprietary dataset belonging to our collaborators, we are unable to disclose it at present.
